# Positive Airway Pressure at Extubation Minimizes Subglottic Secretion Leak In Vitro

**DOI:** 10.3390/jcm11020307

**Published:** 2022-01-08

**Authors:** Tzu-Pei Wang, Hsin-Hsien Li, Hui-Ling Lin

**Affiliations:** 1 Division of Respiratory Therapy, Department of Chest Medicine, Taipei Veterans General Hospital, Taipei 11217, Taiwan; tpwang2@vghtpe.gov.tw; 2 Department of Respiratory Therapy, College of Medicine, Chang Gung University, Taoyuan 33302, Taiwan; hsinhsien@mail.cgu.edu.tw; 3 Institute of Emergency and Critical Care Medicine, School of Medicine, National Yang Ming Chiao Tung University, Taipei 11221, Taiwan; 4 Department of Respiratory Care, Chang Gung University of Science and Technology, Chiayi 61363, Taiwan

**Keywords:** extubation, continuous positive airway pressure, suction, subglottic secretion, viscosity

## Abstract

Accumulated secretion above the endotracheal tube cuff can be aspirated during extubation after deflation. The possible techniques for minimizing pulmonary aspiration from subglottic secretion during extubation have not been well explored. This study aimed to determine the effect of different extubation techniques on secretion leakage. An endotracheal tube was placed in a tube mimicking an airway. We measured the leak volume of water or artificial sputum of different viscosities with three extubation techniques—negative pressure with suctioning; positive pressure with a resuscitator; and continuous positive airway pressure set at 5, 10, and 20 cm H_2_O. Extubation with continuous positive airway pressure resulted in lower secretion leakage than that with negative pressure with suctioning and positive pressure with a resuscitator. Increasing the continuous positive airway pressure level decreased secretion leakage volume during extubation. We further determined a correlation of leak volume with sputum viscosity. Continuous positive airway pressure at 5 cm H_2_O produced lower volume secretion leakage than the other two techniques, even with higher secretion viscosity. Based on these results, using continuous positive airway pressure with a previous ventilator continuous positive airway pressure/positive end-expiratory pressure setting for extubation is recommended.

## 1. Introduction

The daily average amount of subglottic secretion, produced below the vocal chords and above the endotracheal tube (ETT) cuff, is approximately 15 mL/day [1]. Subglottic secretion accumulates above the ETT cuff when inflated and cannot be eliminated without a special suction tool. Extubation, the process of removing an ETT, is crucial for patients receiving mechanical ventilation support [2]. During the extubation process, once the ETT cuff is deflated, accumulated secretions can be aspirated to the lower airways and lung periphery [3].

The technical aspects of subglottic secretion elimination during extubation have not been well explored [4]. Generally, two techniques developed to minimize pulmonary aspiration from subglottic secretions are used for extubation—positive or negative pressure [3,5,6]. The negative pressure extubation technique applies continuous endotracheal suctioning while the ETT is removed from the upper airway. Continuous suctioning is purposely applied to extract subglottic secretions after deflating the cuff. In contrast, the positive pressure extubation technique applies an end-expiratory positive pressure while removing the ETT. The gas flow generated by positive pressure pushes subglottic secretions toward the laryngopharynx and oropharynx [7]. This positive pressure can be generated by squeezing the resuscitator or from the ventilator.

The extubation technique affects the amount of subglottic secretion reaching the lower airways. According to a survey on extubation practices in the United Kingdom, most critical care nurses applied tracheal suctioning during cuff deflation at extubation. A similar clinical practice has been found in Argentina [4,8]. However, the impact of the positive pressure extubation technique on secretion leakage volume has not been well explored for a recommended pressure level. Hodd et al. compared the amount of section leakage to the lower airways between the negative and positive pressure extubation techniques [3]. They demonstrated that the volume of water aspirated was lower with positive pressure when applying the ventilator’s continuous positive airway pressure (CPAP) mode and that the pressure level was negatively correlated with the volume of water leakage. Similarly, Andreu applied the pressure support mode of a mechanical ventilator at extubation and showed a lower volume of water leakage than that with the application of negative pressure [5].

Pathophysiology may alter mucus properties [9]. Patients with acute exacerbation of chronic obstructive pulmonary disease (AECOPD) tend to produce thin, highly elastic mucus, whereas those with pulmonary pneumonia tend to have higher viscosity [10]. In patients with cystic fibrosis, sodium absorbance is increased and chloride and bicarbonate are eliminated in the epithelium of the upper airways, leading to dehydration and increased viscosity [9]. Mucus viscosity is a factor that affects secretion leakage volume toward the lower airways at extubation as lower mucus viscosity increases distance the mucus can flow. This study aimed to determine the effect of different extubation techniques on secretion leakage of different viscosities.

## 2. Materials and Methods

### 2.1. Tracheal Model

**Figure 1:**
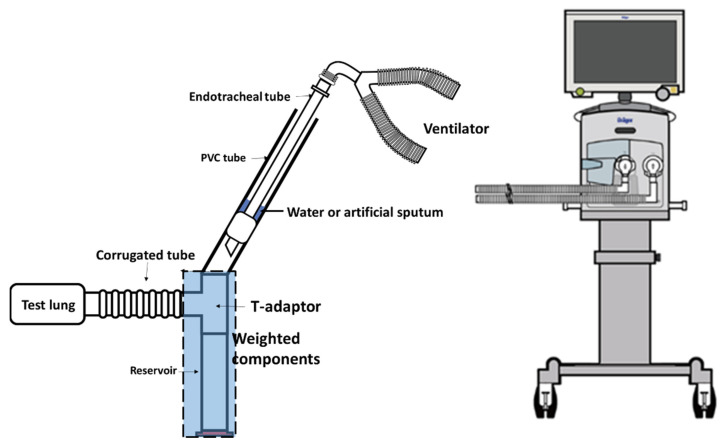
Apparatus used for the tracheal model. An endotracheal tube was placed in the center of a PVC tube (as a tracheal airway) and then connected to a T-adaptor to a reservoir. A ventilator was connected to the proximal of the endotracheal tube. The cuff was inflated with 10 mL of water or artificial sputum. The T-adaptor and the reservoir were weighted after each experiment.

A tracheal model from a previous study was modified [5]. A 22 mm polyvinylchloride (PVC) tube was fixed at a 60° angle to simulate the tracheal airway of an adult patient in Flower’s position, which may have maximum impact on mucus flow toward the lower airways due to gravity [11]. A 7.5 mm ETT (Teleflex Corp., Pennsylvania, USA) with cuff inflation was placed in the center of the tracheal airway. The tracheal airway was first filled with colored water to ensure proper cuff inflation without water leakage, and 15 mL of air was required to fully inflate the cuff. The distal end of the tracheal airway was connected to a T-adaptor. One end of the T-adaptor was connected to a reservoir, and the other was connected to a passive test lung (Siemens Healthcare GmbH, Erlangen Germany). Figure 1 shows the apparatus used for the tracheal model. A ventilator (Drager AG & Co., Lubeck, Germany) was attached to the proximal end of the ETT. The test lung was placed at 90 degrees from the T-adaptor; thus, fluid was less likely to enter the test lung due to gravity.

To ensure each component of the tracheal model were tried prior to the experiment, the ETT, the T-adaptor, the reservoir, and the PVC tube were washed and air dried, and a new corrugated tube was used for each run.

### 2.2. Preparation of Artificial Sputum

To evaluate the influence of mucus viscosity on liquid leakage during extubation, two artificial mucus preparations were used. Two types of artificial mucus, a mixture of porcine mucin (Sigma-Aldrich Co. LLC, Missouri, USA) and water, were prepared—40 mg/mL (sputum 1, viscosity: 5.79 ± 0.09 mPas) and 80 mg/mL (sputum 2, viscosity: 9.45 ± 0.04 mPas). The sputum viscosities aimed to imitate the characteristics of sputum produced by patients with AECOPD (sputum 1) and pneumonia (sputum 2) according to the clinical judgement of a respiratory therapist with 10 years of experience. Additionally, a set of experiments with water instead of artificial mucus was performed.

### 2.3. Extubation Techniques

The PVC tube was first filled with 10 mL of liquid, which was trapped above the ETT cuff and presented as subglottic secretion. Then, three extubation techniques were applied. Extubation techniques designated for comparison are described in Table 1. In each case, the cuff of the ETT was deflated by retrieving the prefilled 15 mL air. The volume of fluid leakage from cuff deflation to the reservoir and remaining in the T-adaptor was determined by weighing the apparatus before and after extubation. Experiments using the three methods with artificial secretion and with water (viscosity: 1 mPa s) were repeated five times for comparison.

### 2.4. Statistical Analysis

Statistical analyses were performed using R programming (R-Studio, Boston, MA, USA). The weight of fluid leakage is expressed as mean ± standard deviation (SD). Comparisons of the extubation technique in terms of leakage volume and viscosity of the mucus were performed using one-way analysis of variance with Scheffe post hoc tests. Pearson’s correlation coefficient was used to determine the correlation between the CPAP level and leakage volume. Differences were considered statistically significant at *p* < 0.05.

## 3. Results

### 3.1. Influence of the Extubation Technique

**Figure 2:**
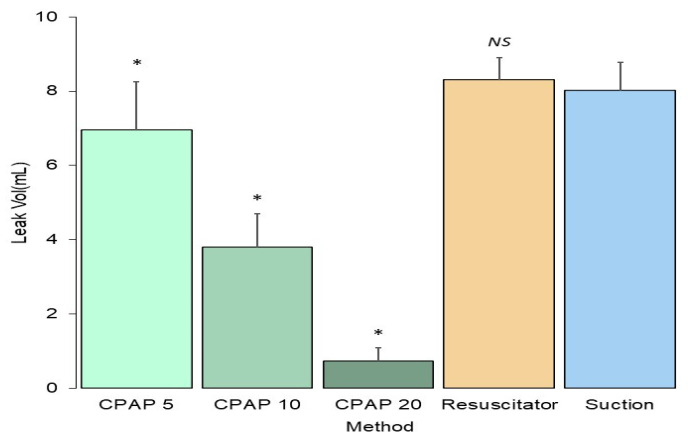
Volume of three types of aspirated fluid with the positive and negative technique. Extubation with continuous positive airway pressure produces lower secretion leakage. P values were determined by one-way analysis of variance with Scheffe post hoc tests as * *p* < 0.001 compared with suction group. CPAP, continuous positive airway pressure.

The leakage volume of fluid with three types tested was significantly lower with the CPAP technique (3.83 ± 2.72 mL) than with the positive pressure technique using a resuscitator (8.31 ± 0.60 mL) or the negative pressure technique (8.01 ± 0.77 mL), as shown in Figure 2. No difference in fluid leakage volume was found between the positive pressure technique using a resuscitator and the negative pressure technique (*p* = 0.78). Increasing the CPAP level significantly decreased fluid leakage volume during extubation (*p* < 0.001). The leakage volumes were 6.96 ± 1.30, 3.79 ± 0.91, and 0.73 ± 0.36 mL for CPAP at 5, 10, and 20 cm H_2_O, respectively.

### 3.2. Influence of Secretion Viscosity

With the negative pressure technique (Table 2), the volume was the highest with water (8.85 ± 0.31 mL, *p* < 0.001), followed by sputum 2 (7.86 ± 0.45 mL) and sputum 1 (7.34 ± 0.54 mL). Similar results were found with the positive pressure technique using a resuscitator (8.96 ± 0.34 mL for water, 7.98 ± 0.34 mL for sputum 1, and 8.00 ± 0.49 mL for sputum 2, *p* < 0.001).

### 3.3. Correlation between Secretion Viscosity and CPAP Level

**Figure 3:**
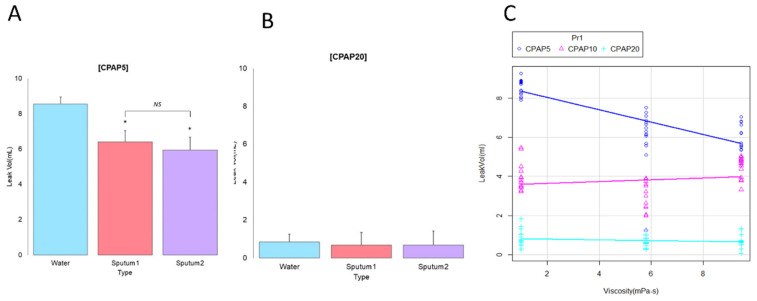
Comparison of mucus viscosity with different extubation techniques. The fluid volume of sputum 1 and 2 is lower than that of water in (**A**) CPAP mode set at 5 cm H_2_O, but there is no difference between the two mucin viscosities at (**B**) a CPAP of 20 cm H_2_O. (**C**) Fluid volume is inversely correlated with viscosity with a CPAP at 5 cm H_2_O, but with a CPAP at 10 and 20 cm H_2_O, the influence of viscosity was minimal. P values were determined by one-way analysis of variance with Scheffe post hoc tests as * *p* < 0.001 compared with water. CPAP, continuous positive airway pressure.

The fluid leak volume was higher with water than with sputums 1 and 2 (Figure 3A; *p* < 0.001), but no difference was observed between sputum 1 and sputum 2 under CPAP at 5 cm H_2_O. The minimum fluid volume (Figure 3B) was 0.84 ± 0.43 mL for water, 0.68 ± 0.31 mL for sputum 1 (*p* = 0.43), and 0.68 ± 0.33 mL for sputum 2 (*p* = 0.436). Fluid viscosity had less impact on leak volume when the CPAP was set at 20 cm H_2_O. The trend of fluid leakage volume according to the three CPAP settings and three fluid viscosities can be seen in Figure 3C. In the CPAP mode, fluid leakage volume was inversely correlated with a CPAP level of 5 cm H_2_O (R^2^ = 0.92, 99% confidence interval: −0.94 to −0.92, *p* < 0.001). Fluid leakage volume was inversely correlated with viscosity at a CPAP of 5 cm H_2_O; however, at a CPAP of 10 and 20 cm H_2_O, the influence of viscosity was minimal.

## 4. Discussion

During extubation, accumulated secretions can be aspirated to the lower airways and can induce further cough reflex or microaspiration. Our results demonstrated that continuous pressure under the CPAP mode minimized the leakage of fluid accumulated above the ETT cuff. Additionally, the secretion viscosity had a minimal impact for a CPAP level of >10 cm H_2_O.

Data on using either positive or negative pressure for extubation are controversial. Some studies have reported the benefits of the positive pressure technique in improving oxygenation and lowering complications, while another study has reported no significant differences between techniques [12,13,14]. Pulmonary aspiration during extubation is one of the most common complications. To minimize this risk, pre-extubation preparations, including pre-suctioning, placing the patient in Fowler’s position, and performing the procedure 1–2 h after a meal, are commonly performed [15]. Airway secretions can be removed by suctioning through the oral cavity and the ETT. However, removing the accumulated subglottic secretion in an intubated patient is challenging, often causing pulmonary aspiration with cuff deflation during extubation [3,6,16]. A previous study revealed that pulmonary aspiration occurred in 68.8% of patients during laryngoscopy and extubation procedures and that the associated complications might lead to cough, wheeze, or declined oxygen saturation [17].

Our study demonstrated that the fluid leakage volume from cuff deflation was not significantly different between the negative pressure technique and the positive pressure technique using a resuscitator, but it was lower using the CPAP technique. The CPAP technique was associated with a minimal decrease in fluid leakage, similar to that reported by Hodd et al. [3]. However, the leakage volume was higher than that reported by Andreu, probably due to the different study design [5]. We established a positive technique using a resuscitator, but not using a CPAP of 0 cm H_2_O, because the use of a resuscitator is more common than adjusting positive end-expiratory pressure (PEEP) settings in clinical practice [8]. In our study, fluid leakage volume was lower with a CPAP of 20 cm H_2_O than with a resuscitator. The positive pressure generated by a resuscitator appears to be intermittent as pressure only occurs when the bag is manually squeezed, and the fluid leaked into the collecting reservoir when the resuscitator was released during exhalation. A previous study compared the fluid volume aspirated with different extubation techniques including CPAP, pressure support ventilation (PSV), and negative pressure techniques [5]. The study results showed that extubation with a PSV/PEEP set at 15/0 cm H_2_O removed 91% (9.1 mL) of the fluid, while only 4% (0.4 mL) of the fluid remained trapped with a CPAP mode set at 15 cm H_2_O. Andreu et al. found that applying a high positive pressure without endotracheal suctioning during cuff deflation reduced the pressure gradient between the airway model system and atmosphere pressure, which would tend to propel sections outward [13]. Gentile et al. described an alternative method to clean subglottic sections by utilizing positive-pressure lung hyperinflation with an inspiratory hold [7]. The gas flow generated by the positive pressure pushes subglottic secretions toward the laryngopharynx and oropharynx. This maneuver was suggested to purge the subglottic secretion during extubation [18]. Therefore, we consider that the leak can be minimized by the sustained positive pressure created by a constant flow, thus offsetting the gravity effect and preventing the secretion from reaching lower positions.

Our study showed that extubation using the CPAP mode greatly reduced fluid leakage volume, and it was inversely correlated with the set CPAP level. Similarly, Hood et al. found a negative correlation between PEEP level and leak volume during extubation [3]. Additionally, a CPAP of 5 cm H_2_O produced lower fluid leakage than the negative pressure technique or the positive pressure technique using a resuscitator, reducing the volume to <1 mL at 20 cm H_2_O. While a previous study recommended using a PEEP of 35 cm H_2_O, our study showed that a CPAP of 5 cm H_2_O alone reduced fluid leakage volume compared to that with the negative and positive pressure techniques [3]. However, high CPAP or PEEP may compromise cardiac contractility and increase pre-/after-loads [19]. Although a CPAP of 20 cm H_2_O minimized secretion leak to <1 mL, it should be applied cautiously, and signs of cardiac compromise should be continuously observed. In any event, we recommend using the CPAP/PEEP setting prior to extubation.

Secretion viscosity influences airway clearance efficiency; thus, it may also influence the amount of secretion aspirated during extubation. A fluid with lower secretion viscosity can more easily leak through the inflated ETT cuff and contribute to the development of ventilator-associated pneumonia [1]. Otherwise, higher secretion viscosity may be retained above the ETT cuff and possibly occlude the side hole. Regarding the influence of mucus viscosities, our results demonstrated that secretion viscosity had a slight impact on fluid gravitational flow to lower positions. However, this influence was offset by a higher constant pressure in the closed system. The propelling forces of the mucus also depend on ventilator settings and lung compliance [20]. The presence of intrinsic PEEP (auto-PEEP) is a common characteristic in mechanically ventilated patients with chronic obstructive pulmonary disease and a long expiratory time constant. Auto-PEEP generates expiratory resistance and causes a relatively lower expiratory flow to move the mucus out of the lung. This suggests that airway clearance seems more important before extubation as auto-PEEP cannot help mucus move to the large airway. A higher PEEP can extend the peripheral airways during expiration and overcome the intrinsic PEEP. Extubation with a higher PEEP setting may be helpful in patients with a high intrinsic PEEP.

Extubation with a deflated cuff is routinely carried out in order to minimize laryngeal injury from an inflated cuff during the removal process. Shamsai first shared his experience of removing the ETT with a “not-overinflated” cuff [21]. The rationale for extubation with a cuff partially inflated was to prevent micro-aspiration from the section above the cuff, which may lead to potential harm, such as aspiration pneumonia. Vance et al. investigated the effect of extubation with the ETT cuff inflated vs. deflated on endotracheal fluid volume in dog cadavers [22]. The post-extubation radiographs showed more residual intratracheal contents with a deflated ETT cuff than with an inflated cuff. Priebe supported the practice of extubation with an inflated cuff to reduce the risk of pulmonary complications post-extubation [23]. This practice with an inflated cuff must be monitored to avoid laryngeal injury. However, there is no strong evidence to support extubation with an inflated cuff to alter the current practice. Our study provides clinicians with more information regarding commonly performed techniques. This study has several limitations owing to the steady nature of a bench study setting. Clinically, a patient receiving extubation with the suctioning technique would generate a cough at extubation, and the aspirated fluid may be coughed out [8]. The simulation in our study could not imitate this cough effect. Additionally, a previous study showed a daily median of 14 (8–28) mL of subglottic secretions with individual variations [24]. Patients undergoing extubation may produce less secretion than expected. Finally, the tested sputum viscosity was based on observation. However, the sputum viscosity produced by patients may vary. Therefore, further clinical studies are warranted.

## 5. Conclusions

Compared to extubation with suctioning or resuscitator use, extubation with the CPAP mode led to a lower fluid leakage from the deflated cuff. A CPAP at 5 cm H_2_O produced a lower fluid leakage volume than the other two techniques. Higher secretion viscosity could slightly lower the leaked fluid volume with suctioning or resuscitator use; sputum viscosity was inversely correlated with fluid leakage volume. However, the phenomenon was minimized when the CPAP level was >10 cm H_2_O.

## Figures and Tables

**Table 1:** Description of extubation procedures.

Method	Description of Procedures
CPAP 5	Ventilator was connected to the ETT, and the ventilator was set at CPAP mode of 5 cm H_2_O. The cuff was deflated after 3 spontaneous breaths, and then the ETT was removed immediately.
CPAP 10	Ventilator was connected to ETT and the ventilator was set at CPAP mode of 10 cm H_2_O. The cuff was deflated after 3 spontaneous breaths, and then the ETT was removed immediately.
CPAP 20	Ventilator was connected to the ETT, and the ventilator was set at CPAP mode of 20 cm H_2_O. The cuff was deflated after 3 spontaneous breaths, and then the ETT was removed immediately.
Resuscitator	A resuscitator was attached to the ETT and then squeezed 3 breaths every 3 s after achieving a pressure of 20 cm H_2_O. The ETT cuff was deflated while squeezing the resuscitator, and then the ETT was removed immediately.
Suction	A 14-Fr suction catheter was inserted into the ETT up to 2 cm above the ETT tip. A wall suction regulator at a pressure of 150 cm H2O was used for continuous suction. The ETT and suction catheter were removed together immediately after the cuff was deflated.

CPAP, continuous positive airway pressure. ETT, endotracheal tube.

**Table 2:** Comparison of mucus viscosity between the different extubation techniques.

	Water	Sputum 1	Sputum 2	* p *
Suction	8.85 ± 0.31	7.34 ± 0.54	7.8 6 ± 0.45	<0.001
Resuscitator	8.96 ± 0.34	7.98 ± 0.34	8.00 ± 0.49	<0.001

## Data Availability

The datasets used and/or analyzed during the current study are available from the corresponding author on reasonable request.
